# Diagnostic Strategy of Early Stage Pancreatic Cancer via Clinical Predictor Assessment: Clinical Indicators, Risk Factors and Imaging Findings

**DOI:** 10.3390/diagnostics12020377

**Published:** 2022-02-01

**Authors:** Ryota Sagami, Takao Sato, Kazuhiro Mizukami, Mitsuteru Motomura, Kazuhisa Okamoto, Satoshi Fukuchi, Yuichiro Otsuka, Takashi Abe, Hideki Ono, Kei Mori, Kurato Wada, Tomoyuki Iwaki, Hidefumi Nishikiori, Koichi Honda, Yuji Amano, Kazunari Murakami

**Affiliations:** 1 Department of Gastroenterology, Oita San-ai Medical Center, 1213 Oaza Ichi, Oita 870-1151, Japan; sagami1985@yahoo.co.jp (R.S.); iwataiwata18@yahoo.co.jp (T.S.); nikki@san-ai-group.org (H.N.); 2 Department of Gastroenterology, Faculty of Medicine, Oita University, 1-1 Idaigaoka, Hasamacho, Yufu 879-5593, Japan; kokamoto@oita-u.ac.jp (K.O.); kei806@oita-u.ac.jp (K.M.); hondak@oita-u.ac.jp (K.H.); murakam@oita-u.ac.jp (K.M.); 3 Department of Gastroenterology, Oita Red Cross Hospital, 3-2-37 Chiyo-Machi, Oita 870-0033, Japan; mmotomura@oita-u.ac.jp; 4 Department of Gastroenterology, Oita City Medical Association Almeida Memorial Hospital, 1509-2 Miyazaki, Oita 870-1195, Japan; satofukuti@yahoo.co.jp (S.F.); kura-wada@oita-u.ac.jp (K.W.); 5 Department of Gastroenterology, Oita Medical Center, 2-11-45 Yokota, Oita 870-0263, Japan; yotsuka78080@gmail.com; 6 Department of Gastroenterology, Oita Kouseiren Tsurumi Hospital, 4333 Tsurumi, Beppu 874-8585, Japan; takashi0315@oita-u.ac.jp; 7 Department of Gastroenterology, Oita Prefectural Hospital, 2-8-1 Bunyo, Oita 870-8511, Japan; spgh6299@dune.ocn.ne.jp; 8 Department of Endoscopy, Urawa Kyosai Hospital, 3-15-31 Harayama, Saitama 336-0931, Japan; tomoyukiiwaki1984@gmail.com (T.I.); amanoyj@gmail.com (Y.A.)

**Keywords:** early diagnosis, high-grade pancreatic intraepithelial neoplasia, pancreatic cancer, pancreatic cancer in situ, pancreatic ductal adenocarcinoma, risk factor, screening, symptom

## Abstract

Early detection of pancreatic ductal adenocarcinoma (PDAC) in the general population is difficult due to unknown clinical characteristics. This study was conducted to clarify the factors associated with early stage PDAC. Well-known symptoms and factors associated with PDAC were classified into clinical indicators, risk factors, and imaging findings concomitant with early stage PDAC. To analyze these factors for the detection of patients with early stage PDAC compared to patients without PDAC, we constructed new diagnostic strategies. The factors of 35 patients with early stage PDAC (stage 0 and IA) and 801 patients without PDAC were compared retrospectively. Clinical indicators; presence and number of indicators, elevated pancreatic enzyme level, tumor biomarker level, acute pancreatitis history, risk factors; familial pancreatic cancer, diabetes mellitus, smoking history, imaging findings; presence and number of findings, and main pancreatic duct dilation were significant factors for early stage PDAC detection. A new screening strategy to select patients who should be examined by imaging modalities from evaluating clinical indicators and risk factors and approaching a definitive diagnosis by evaluating imaging findings had a relatively high sensitivity, specificity, and areas under the curve of 80.0%, 80.8%, and 0.80, respectively. Diagnosis based on the new category and strategy may be reasonable for early stage PDAC detection.

## 1. Introduction

Although pancreatic ductal adenocarcinoma (PDAC) has a poor prognosis with a 5 year overall survival rate of <10% [1], those of Union for International Cancer Control (UICC) stages 0 and IA PDAC are relatively high, at 85.8% and 68.7%, respectively [2]. Therefore, stages 0 and IA PDACs are ideal targets for early stage PDAC detection [3,4]. However, patients with UICC stages 0 and ⅠA PDAC account for only 1.7% and 4.1% of overall patients with PDAC, respectively [2]. Screening for PDAC in the general population is not recommended because of the low incidence of PDAC (approximately 0.013% per year) [5,6,7]. Therefore, surveillance efforts for PDAC in asymptomatic high-risk patients with PDAC-associated gene mutations or familial pancreatic cancer are gradually increasing worldwide [8,9]. However, only 10% or less patients with PDAC have familial risks or inherited genetic syndromes, and the diagnostic rate based on surveillance is not high for early stage PDAC [10]. For early stage PDAC detection, systematic screening of the general population is needed.

The Japanese Pancreas Society recommends a wide and comprehensive screening for individuals with clinical symptoms and risk factors, including familial risks associated with PDAC, and these factors can be determined using general questionnaires and blood tests [11,12]. However, imaging findings are only clarified when various imaging examinations are performed. In addition, only approximately 30% of early stage PDAC is undetectable as a tumor using various imaging modalities [4,13,14,15]. Therefore, indirect imaging findings, such as main pancreatic duct (MPD) dilation or pancreatic cysts, including intraductal mucinous cystic neoplasm (IPMN), are important diagnostic signs [13,14,16,17,18]. Realistically, examining all individuals for factors associated with PDAC in detail is difficult, and not all of them can be examined by imaging modalities. In addition, only 25% of patients with early stage PDAC show any clinical symptoms [15], and selection of individuals who should be screened for multiple factors is needed. This study aimed to classify well-known clinical symptoms, risk factors, and indirect imaging findings associated with PDAC to establish a new screening strategy for detecting early stage PDAC in daily outpatients.

## 2. Materials and Methods

### 2.1. Eligibility Criteria

Between December 2014 and July 2019, patients with UICC stages 0 and IA PDAC (invasive PDAC with tumor diameter ≤20 mm localized within the pancreas, without regional lymph nodes and distant metastasis) that were histopathologically diagnosed from surgically resected specimens at eight participating institutions were included. In this study, UICC stage 0 PDAC included only high-grade pancreatic intraepithelial neoplasm (PanIN)-3 [2,19,20], and were defined as early stage PDAC. Patients with high-grade IPMN, invasive cancer derived from IPMN, and recurrent PDAC were excluded. During the same period, patients in whom PDAC and other malignancies were completely ruled out by endoscopic ultrasound (EUS) with detailed preoperative questionnaires and blood tests were defined as patients without PDAC. Even when patients’ pancreatic tumors were suspected to be PDAC, they were also enrolled in the group without PDAC when the lesion was histologically diagnosed to be non-malignant by EUS-guided fine needle aspiration biopsy. In addition, this suggests a benign condition, when its form did not change in various imaging modalities over a 2 year period.

The protocol of this retrospective multicenter study was approved by the institutional review board (IRB no. 019002 K), and the need for informed consent was waived because of the retrospective nature of this study.

### 2.2. Clinical Indicators, Risk Factors, and Imaging Findings Associated with Pancreatic Ductal Adenocarcinoma (PDAC)

In this study, clinical symptoms concomitant with PDAC and factors associated with PDAC were classified into three categories. First, factors that can be identified from general questionnaires and blood tests in clinical practice were classified as “clinical indicators” and “risk factors”. Clinical indicators included clinical symptoms, medical histories, or changes in blood test results that may be caused by the onset of PDAC. Clinical indicators are the first clue to suspecting the existence of PDAC. Risk factors included medical histories or preferences that may be the cause of PDAC onset.

Second, the imaging findings mainly by EUS were also evaluated. However, early stage PDAC is often undetectable as a tumor. Therefore, “imaging finding” was defined as the tumor itself and an important indirect imaging finding for detecting early stage PDAC using various imaging modalities, including EUS.

On the one hand, clinical indicators included abdominal pain, nausea, back pain, weight loss, jaundice, new-onset or worsening type 2 diabetes mellitus (DM), elevated pancreatic enzyme (amylase) level, elevated tumor biomarker (carbohydrate antigen 19-9 (CA19-9), carcinoembryonic antigen (CEA)) levels, and medical history of acute pancreatitis [5,11,21,22,23]. On the other hand, risk factors included sporadic family history of pancreatic cancer, familial pancreatic cancer, hereditary pancreatic cancer syndrome/hereditary pancreatitis, DM, chronic pancreatitis, heavy alcohol consumption, smoking history, and obesity [5,21,22,23,24]. Imaging findings included the tumor itself and indirect imaging findings (MPD dilation, pancreatic cyst, and IPMN) [11,14,16,17,25,26,27,28,29].

**Figure 1:**
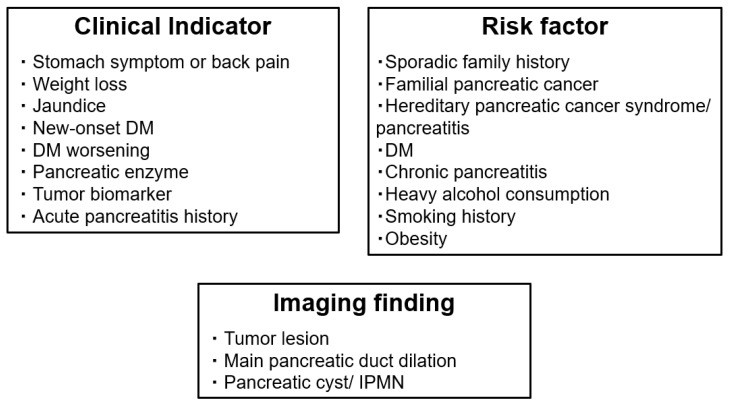
Newly classified factors associated with early stage PDAC detection: clinical indicators, risk factors, and indirect imaging findings. Well-known factors associated with PDAC were newly classified into three categories: clinical indicators (may be caused by PDAC onset), risk factors (may be the cause of PDAC onset), and imaging findings are concomitant with early PDAC. DM, diabetes mellitus; IPMN, intraductal papillary mucinous neoplasm; PDAC, pancreatic ductal adenocarcinoma.

These factors have been suggested as suggestive of early stage PDAC detection in many guidelines and reviews [5,11,14,21,22,24]. These three new categories and previously reported percentage of each factor in patients with stage 0–Ⅰ PDAC is shown in Figure 1 and Table 1 [15,26].

In each category, each factor, the presence or absence of factors, and the number of factors were evaluated.

### 2.3. Study Outcomes

The primary endpoint was the usefulness of clinical indicators, risk factors (questionnaires and blood tests), and imaging findings for the detection of patients with early stage PDAC compared to patients without PDAC. The secondary endpoint was a new strategy established for the selection of individuals who should be screened by imaging modalities for detecting early stage PDAC and to evaluate the diagnostic reliability of the new strategy.

### 2.4. Statistical Analyses

Individual factors in the three categories, and presence and number of each of the three categories were compared between patients with early stage PDAC and patients without PDAC. Continuous variables are expressed as mean ± standard deviation because they are normally distributed. Categorical data were compared using the chi-squared test or Fisher’s exact test. Logistic regression analyses were performed on covariates that could potentially influence the detection of patients with early stage PDAC. Factors that were significant in the univariate analysis were included in the multivariate logistic regression analyses to clarify the most significant factor for the diagnosis of patients with early stage PDAC. Statistical significance was set at *p* < 0.05. Sensitivity and specificity were evaluated using two-way contingency tables and receiver operating characteristic curves. The area under the curve (AUC) was calculated using a 95% confidence interval (CI). All data analyses were performed using the Statistical Package for the Social Sciences (version 28.0; IBM Corp, Armonk, NY, USA).

## 3. Results

Overall, 35 patients with early stage PDAC, including 6 with stage 0 PDAC and 29 with stage IA PDAC, and 801 patients without PDAC were included in this study. All early stage PDACs were detected as mass lesions or on the basis of indirect imaging findings (MPD dilation and pancreatic cyst) by EUS and surgical resection. The median histopathological tumor diameter of stage IA PDAC was 13 (range, 3–18) mm, and PDACs were located in the head and body-tail in 34% and 66% of patients, respectively.

A comparison of each factor in patients with early stage PDAC and those without PDAC is shown in Table 2.

In clinical indicators, individual factors, such as elevated pancreatic enzyme level, elevated tumor biomarker level, acute pancreatitis history, and the presence and number of these indicators, were significantly associated with the diagnosis of patients with early stage PDAC compared to patients without PDAC (*p* = <0.001, 0.001, <0.001, 0.009, and <0.001, respectively). The presence and number of risk factors were not identified as significant factors; however, individual factors such as familial pancreatic cancer, DM, and smoking history were significant factors (*p* = 0.013, 0.044, and <0.001, respectively) in the detection of early stage PDAC. In multivariate logistic regression analysis of significant clinical indicators and risk factors, elevated pancreatic enzyme level, acute pancreatitis history, familial history of pancreatic cancer, and smoking history were proven to be the most significant factors in the detection of early stage PDAC (*p* = 0.002, 0.001, 0.043, and 0.011, respectively) (Table 3).

In imaging findings, tumor, MPD dilation, and presence and number of imaging findings were significant factors (*p* <0.001, <0.001, <0.001, and <0.001, respectively) (Table 2).

The presence, number, and specific individual factors of clinical indicators (elevated pancreatic enzyme level, tumor biomarker level, and acute pancreatitis history) were significantly associated with the detection of early stage PDAC. Although the presence and number of risk factors were not significant, three significant risk factors—familial pancreatic cancer, DM, and smoking history—were. Considering these results and recent consensus, we find that imaging examination may be strongly recommended in individuals with two or more clinical indicators (according to the level of significance of the number of clinical indicators), one significant indicator, or one clinical indicator with one significant risk factor. Imaging examination can also be strongly recommended for patients with no clinical indicators but with one significant risk factor of familial pancreatic cancer. However, not all the patients with smoking history or DM who had no clinical indicator should be screened. Therefore, imaging examination may also be partially recommended for patients with no clinical indicators but with one significant risk factor of DM or a smoking history. Imaging examinations can be recommended for patients with one significant indicator, without significant risk factors.

**Figure 2:**
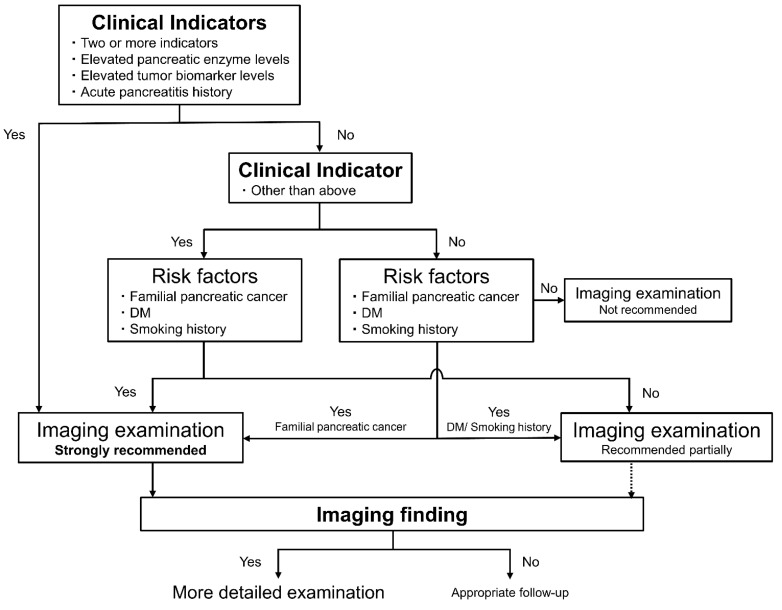
Newly constructed step-by-step diagnostic strategy for early stage PDAC evaluating clinical indicators, risk factors, and indirect imaging findings in outpatients. Considering the significant factors for early stage PDAC detection, we found that imaging examination can be strongly recommended in individuals with two or more clinical indicators, one significant indicator, or one clinical indicator with one significant risk factor. For patients with no clinical indicators but with one significant risk factor of familial pancreatic cancer, imaging examination may also be strongly recommended. DM, diabetes mellitus; IPMN, intraductal papillary mucinous neoplasm; MPD, main pancreatic duct; PDAC, pancreatic ductal adenocarcinoma.

As shown in Figure 2, determining whether imaging evaluation is necessary to evaluate clinical indicators and risk factors is recommended.

In these selected patients examined by imaging modality, the diagnostic reliability of early stage PDAC detection was elevated. When all the patients for whom imaging evaluation were strongly recommended according to the strategy were examined, the sensitivity, specificity, and AUC for early stage PDAC detection were 80.0%, 53.3%, and 0.67 (95% CI, 0.58–0.75), respectively. In addition, when the patients for whom imaging evaluations were strongly recommended according to the strategy had one or more imaging findings, the sensitivity, specificity, and AUC were 80.0%, 80.8%, and 0.80 (95% CI, 0.73–0.88), respectively.

## 4. Discussion

### 4.1. Diagnostic Strategy for Early Stage PDAC Detection in Outpatients

In this study, many factors associated with PDAC were newly classified into three categories, namely, clinical indicators, risk factors, and imaging findings, to compare the factors of patients with early stage PDAC and without PDAC. In the daily outpatient clinic, most patients complain of any symptoms or changes in medical conditions, including clinical indicators. In this analysis, the effect of the presence and number of clinical indicators (especially elevated pancreatic enzyme level and tumor biomarker level and acute pancreatitis history were significant) were clarified. Although the presence and number of risk factors were not significantly associated, three significant risk factors, namely, familial pancreatic cancer, DM, and smoking history, were identified. The presence and number of imaging findings were significant factors in the diagnosis of early stage PDAC. Comprehensively, imaging examination can be strongly recommended in individuals who meet the shown strategy (Figure 2), and the diagnostic ability was relatively high. In outpatients, patients selected on the basis of these clinical indicators and risk factors, which can be obtained easily by questionnaires and blood tests, should undergo imaging examination according to this strategy. In addition, patients with tumors and those with indirect imaging findings should be examined in more detail. Patients with strongly suspicious PDAC lesions are recommended to be examined using preoperative histopathological diagnostic methods, such as EUS-guided fine needle aspiration biopsy and pancreatic juice cytology [14,30].

### 4.2. Evidence of Significant Clinical Indicators, Risk Factors, and Indirect Imaging Findings for PDAC

PDAC typically develops with few symptoms and is often advanced when symptoms, such as painless jaundice, are present [31]. Abdominal and back pain, weight loss, and jaundice occur in 48–82%, 56–80%, and 66–84% of patients with advanced PDAC, respectively [32]. However, only 25% of patients with early stage PDAC show these symptoms [15]. Commonly atypical symptoms, such as weight loss and abdominal pain, might lead to a delay in diagnosis; therefore, these clinical indicators may be the first reason to consider early stage PDAC diagnosis. Levels of pancreatic enzymes, such as amylase, may increase due to pancreatic duct stenosis following PDAC occurrence, and 20–50% of patients with PDAC show elevated serum pancreatic enzyme level [11]. In contrast, CEA and CA19-9, common tumor biomarkers used in diagnosing pancreatic cancer, have sensitivity and specificity for PDAC of 70–90% and 43–91% and 45–60% and 75–99%, respectively [32,33,34]. Using the combination of the optimal cutoff value of these markers, the positive predictive value for the detection of advanced PDAC is 91% [35]. Although only 56% of patients with stage IA show elevated CA19-9 level [36], the usefulness of pancreatic enzymes and tumor biomarkers in early stage PDAC detection remains controversial.

Acute pancreatitis appearing 2 years before the diagnosis of PDAC is often the result of tumor-related ductal obstruction; therefore, acute pancreatitis may be a clinical manifestation of PDAC [5]. A nationwide matched cohort study showed that the 2 year absolute risk of PDAC among patients with acute pancreatitis is 0.7% (95% CI, 0.6–0.8%). Other studies have shown that PDAC detection rate after acute pancreatitis history is approximately 1%, with the highest being in the first 2 years [37,38]. In contrast, long-standing chronic pancreatitis is recognized as a strong risk factor for PDAC, and the lifetime risk of PDAC is elevated 16.2-fold (95% CI, 12.6–20.7) [39].

Patients with a family history of PDAC have a significantly increased PDAC risk by 1.8-fold (95% CI, 1.5–2.1) [40]. Another study showed that the expected rate of PDAC is significantly elevated in members of familial pancreatic cancer kindreds (9.0; 95% CI, 4.5–16.1), but not in sporadic pancreatic cancer kindreds (1.8; 95% CI, 0.22–6.4) [41]. Patients with hereditary pancreatic cancer and pancreatitis syndrome have a stronger risk of PDAC occurrence [42,43].

Type 2 DM is also closely associated with PDAC; patients with DM have a higher risk of PDAC occurrence compared with individuals without DM (1.9; 95% CI, 1.7–2.3) [44]. The risk of PDAC increased more significantly when patients newly diagnosed with DM experienced recent weight loss (6.8; 95% CI, 4.6–10.0) [45]. In addition, patients with a 1 year or less history of DM have the highest risk of PDAC [44]. DM worsening increases the risk of PDAC in individuals with the highest hemoglobin A1c quartile compared to those with the lowest (2.0; 95% CI,1.4–2.8) [46]. Although the intricate and multidirectional relationship between DM and PDAC is yet to be fully understood, new-onset DM and DM worsening may also be manifestations of PDAC, and long-term DM history may be a risk factor for PDAC [21,47]. Smoking is also closely associated with PDAC occurrence, and the risks of PDAC in current and former smokers are significantly higher than those in non-smokers (1.8; 95% CI, 1.7–1.9 and 1.2, 95% CI, 1.1–1.2, respectively) [48].

Most of the factors mentioned above are mainly analyzed in patients with advanced PDAC, but the data on early stage PDAC are insufficient [15,26]. In this study, a more effective method for early stage PDAC detection was considered, and new categories of clinical indicators and risk factors were compared in patients with and without early stage PDAC. In addition, a new diagnostic strategy for early stage PDAC detection using these categories has been developed. Diagnostic ability is drastically increased by evaluating the imaging findings. Imaging findings, especially the indirect imaging findings of early stage PDAC, are important.

Early stage PDAC, especially stage 0 PDAC (high-grade PanIN), is often undetectable by various imaging modalities as a tumor. In contrast, indirect imaging findings concomitant with early stage PDAC are gradually being revealed [14,15]. MPD dilation is a predictor of PDAC and is often associated with downstream MPD stenosis. The detection and follow-up of MPD dilation, including high-grade PanIN, are necessary for early stage PDAC, including high-grade PanIN [14,17,25]. MPD dilation concomitant with early stage PDAC can be detected in 52–84% of cases [15,16,26,49]. Pancreatic cysts, including retention cysts and branch-duct IPMN, are also considered a risk factor for PDAC [11,17,27,28,29], and long-term follow-up of these cysts contributes to the detection of early stage PDAC [17]. Concomitant PDAC is found in approximately 40% of detected PDAC with branch-duct IPMN [28,29], and high-grade PanIN is detected in 6.3–19% of resected pancreatic specimens of branch-duct IPMNs [50,51,52]. These cysts concomitant with early stage PDAC can be detected in 21–74% of cases [15,16,26,49]. In this study, the presence and number of imaging findings (especially MPD dilation) played an important role in the diagnosis of early stage PDAC.

### 4.3. Future Perspective of Screening for PDAC

Although only asymptomatic patients with PDAC-associated gene mutations or familial pancreatic cancer are only screened worldwide [8,9], only 10% or less patients with PDAC have these risks [5], and the diagnostic rate of these surveillance is low for early stage PDAC [10]. In contrast, some risk prediction models in the general population were proposed, and they evaluated the multiple risks and symptoms associated with PDAC, especially new-onset DM, and could detect patients with PDAC with an AUC of 0.68–0.87 [53,54,55,56]. These studies regarding risk prediction models for PDAC remain unsatisfactory in quality because they do not evaluate early stage PDAC. Whether these models contribute to early stage PDAC remains unknown.

Recent studies on risk prediction models for early stage PDAC detection have shown relatively high detection rates. A scoring system detected PDAC ≤ 20 mm with sensitivity, specificity, and AUC of 100%, 64%, and 0.82, respectively, and the detection rate of small PDAC was 3.4% [23]. The other scoring system showed a detection rate of 6.1% for stage I and II PDACs [57]. A large cohort study showed a detection rate of 0.8% for stages 0 and I in approximately 5000 patients with some risks or symptoms [25]. These studies evaluating multiple risks and symptoms associated with PDAC could detect early stage PDAC with relatively high detection rates. However, they did not evaluate separately clinical data obtained in outpatients (questionnaires and blood sampling), and imaging findings that require additional radiological interventions.

In that respect, this study has the following strengths. This is the first study evaluating the clinical characteristics and imaging findings of patients with early stage PDAC, compared to patients without PDAC. The compared data were newly classified into clinical indicators, risk factors (based on easy questionnaires and blood test), and imaging findings (requiring radiological intervention). This step-by-step diagnostic strategy based on the new category showed relatively high diagnostic ability. This strategy may be ideal for selecting individuals who should be screened and should undergo imaging examination for early stage PDAC. This study has some limitations. The number of patients with early stage PDAC and those without PDAC was limited. In addition, since individuals with symptoms or risk associated with PDAC were screened selectively, there is a possibility of selection bias in the control group. This was a multi-center study; however, it was performed in a single prefecture. Considering the racial and regional differences of the screened group, further confirmation via a nationwide or worldwide prospective comparative multicenter study under the same conditions and with long-term observation is necessary to improve the versatility of the data.

## 5. Conclusions

The presence and number of clinical indicators, especially elevated pancreatic enzyme level, tumor biomarker level, and acute pancreatitis history; risk factors for familial pancreatic cancer, DM, and smoking history; and the presence and the number of imaging findings, especially tumor and MPD dilation, were found to be significant factors for the detection of patients with early stage PDAC compared to patients without PDAC. A newly constructed step-by-step screening strategy to select patients who should be examined by imaging modalities from evaluating clinical indicators and risk factors and approaching a definitive diagnosis by evaluating imaging findings had relatively high diagnostic ability and may be reasonable for early stage PDAC detection in daily outpatients.

## Figures and Tables

**Table 1:** Classification of and each rate of factors associated with PDAC detection: clinical indicators, risk factors, and indirect imaging findings.

		Stage 0–I PDAC Cases (%)
Clinical indicators	Stomach symptoms or back pain	22.9–24.5
	Weight loss	3.1
	Jaundice	0.5–1.0
	New-onset or worsening DM	11.5
	Elevated pancreatic enzyme level	5.8–48.9
	Elevated tumor biomarker level	3.9–26.6
	Acute pancreatitis history	6.5
Risk factors	Sporadic family history	2.4–4.5
	Familial pancreatic cancer	0
	Hereditary pancreatic cancer syndrome/pancreatitis	0
	DM	27.1–32.0
	Chronic pancreatitis	7.4–15.0
	Heavy alcohol consumption	13.0–25.5
	Smoking history	30.5–31.0
	Obesity	3.1–6.5
Imaging findings	Tumor	51.5–76.3
	MPD dilation	77.2–88.4
	Pancreatic cyst/IPMN	26.0–38.0

DM, diabetes mellitus; IPMN, intraductal papillary mucinous neoplasm; MPD, main pancreatic duct; PDAC, pancreatic ductal adenocarcinoma.

**Table 2:** Significant factors in the three categories for the diagnosis of patients with early stage PDAC.

		Early Stage PDAC (*n* = 35)	Without PDAC (*n* = 801)	*p*-Value
Mean age ± SD, y		69.7 ± 9.9	67.2 ± 12.5	0.251
Male sex, *n* (%)		14 (40.0)	465 (58.1)	0.038
Clinical indicators, *n* (%)	Stomach symptoms or back pain	10 (28.6)	230 (28.7)	0.985
	Weight loss	3 (8.6)	130 (16.2)	0.235
	Jaundice	1 (2.9)	9 (1.1)	0.373
	New-onset or worsening DM	7 (20.0)	87 (10.1)	0.100
	Elevated pancreatic enzyme level	13 (37.1)	102 (12.7)	<0.001 *
	Elevated tumor biomarker level	12 (34.3)	110 (13.7)	0.001 *
	Acute pancreatitis history	6 (17.1)	23 (2.9)	<0.001 *
	Presence of clinical indicator	29 (82.9)	478 (59.7)	0.009 *
	Number of clinical indicators ± SD	1.5 ± 1.0	0.9 ± 0.9	<0.001 *
Risk factors, *n* (%)	Sporadic family history	1 (2.9)	102 (12.7)	0.116
	Familial pancreatic cancer	2 (5.7)	6 (0.7)	0.013 *
	Hereditary pancreatic cancer syndrome/pancreatitis	1 (2.9)	0 (0)	1.000
	DM	16 (45.7)	236 (29.5)	0.044 *
	Chronic pancreatitis	1 (2.9)	22 (2.7)	0.969
	Heavy alcohol consumption	7 (20.0)	245 (30.6)	0.187
	Smoking history	18 (51.4)	186 (23.2)	<0.001 *
	Obesity	4 (11.4)	195 (24.3)	0.089
	Presence of risk factor	26 (74.3)	550 (68.7)	0.483
	Number of risk factors ± SD	1.4 ± 1.0	1.2 ± 1.1	0.316
Imaging findings, *n* (%)	Tumor	30 (85.7)	10 (1.2)	<0.001 *
	MPD dilation	28 (80.0)	108 (13.5)	<0.001 *
	Pancreatic cyst/IPMN	16 (45.7)	301 (37.6)	0.333
	Presence of imaging finding	35 (100)	378 (47.2)	<0.001 *
	Number of imaging findings ± SD	2.1 ± 0.7	0.5 ± 0.6	<0.001 *
All factors, *n* (%)	Presence of all factors	35 (100)	754 (94.1)	0.998
	Number of all factors ± SD	5.0 ± 1.9	2.6 ± 1.6	0.363

* *p* < 0.05, compared to patients with small PDAC and without PDAC. DM, diabetes mellitus; IPMN, intraductal papillary mucinous neoplasm; MPD, main pancreatic duct; PDAC, pancreatic ductal adenocarcinoma; SD, standard deviation.

**Table 3:** Most significant factors in clinical indicators and risk factors: factors obtained from easy questionnaires and blood test.

		OR	95% CI	*p*-Value
Clinical indicators, *n* (%)	Elevated pancreatic enzyme level	4.8	1.8–12.5	0.002 *
	Acute pancreatitis history	6.9	2.1–22.0	0.001 *
Risk factors, *n* (%)	Familial pancreatic cancer	6.4	1.1–39.0	0.043 *
	Smoking history	2.6	1.2–5.6	0.011 *

* *p* < 0.05, compared to patients with small PDAC and without PDAC. CI, confidence interval; DM, diabetes mellitus; OR, odds ratio.

## Data Availability

The dataset used during the current study is available from the corresponding author on reasonable request.

## References

[1] Siegel R.L., Miller K.D., Fuchs H.E., Jemal A. (2021). Cancer Statistics, 2021. CA Cancer J. Clin..

[2] Egawa S., Toma H., Ohigashi H., Okusaka T., Nakao A., Hatori T., Maguchi H., Yanagisawa A., Tanaka M. (2012). Japan Pancreatic Cancer Registry; 30th year anniversary: Japan Pancreas Society. Pancreas.

[3] Hur C., Tramontano A.C., Dowling E.C., Brooks G.A., Jeon A., Brugge W.R., Gazelle G.S., Kong C.Y., Pandharipande P.V. (2016). Early Pancreatic Ductal adenocarcinoma survival is dependent on size: Positive Implications for future targeted screening. Pancreas.

[4] Lennon A.M., Wolfgang C.L., Canto M.I., Klein A.P., Herman J.M., Goggins M., Fishman E.K., Kamel I., Weiss M.J., Diaz L.A. (2014). The early detection of pancreatic cancer: What will it take to diagnose and treat curable pancreatic neoplasia?. Cancer Res..

[5] Singhi A.D., Koay E.J., Chari S.T., Maitra A. (2019). Early Detection of Pancreatic Cancer: Opportunities and Challenges. Gastroenterology.

[6] Owens D.K., Davidson K.W., Krist A.H., Barry M.J., Cabana M., Caughey A.B., Curry S.J., Doubeni C.A., Epling J.W., Kubik M. (2019). Screening for Pancreatic Cancer. JAMA.

[7] Aslanian H.R., Lee J.H., Canto M.I. (2020). AGA Clinical Practice Update on Pancreas Cancer Screening in High-Risk Individuals: Expert Review. Gastroenterology.

[8] Henrikson N.B., Aiello Bowles E.J., Blasi P.R., Morrison C.C., Nguyen M., Pillarisetty V.G., Lin J.S. (2019). Screening for Pancreatic Cancer. JAMA.

[9] Vasen H., Ibrahim I., Ponce C.G., Slater E.P., Matthäi E., Carrato A., Earl J., Robbers K., van Mil A.M., Potjer T. (2016). Benefit of surveillance for pancreatic cancer in high-risk individuals: Outcome of Long-term prospective follow-up studies from three European expert centers. J. Clin. Oncol..

[10] Corral J.E., Das A., Bruno M.J., Wallace M.B. (2019). Cost-effectiveness of pancreatic cancer surveillance in high-risk individuals: An economic analysis. Pancreas.

[11] Yamaguchi K., Okusaka T., Shimizu K., Furuse J., Ito Y., Hanada K., Shimosegawa T., Okazaki K. (2017). Clinical Practice Guidelines for Pancreatic Cancer 2016 from the Japan Pancreas Society. Pancreas.

[12] Okusaka T., Nakamura M., Yoshida M., Kitano M., Uesaka K., Ito Y., Furuse J., Hanada K., Okazaki K. (2020). Clinical Practice Guidelines for Pancreatic Cancer 2019 from the Japan Pancreas Society: A Synopsis. Pancreas.

[13] Kogekar N., Diaz K.E., Weinberg A.D., Lucas A.L. (2020). Surveillance of high-risk individuals for pancreatic cancer with EUS and MRI: A meta-analysis. Pancreatology.

[14] Sagami R., Yamao K., Nakahodo J., Minami R., Tsurusaki M., Murakami K., Amano Y. (2021). Pre-operative imaging and pathological diagnosis of localized high-grade pancreatic intra-epithelial neoplasia without invasive carcinoma. Cancers.

[15] Kanno A., Masamune A., Hanada K., Maguchi H., Shimizu Y., Ueki T., Hasebe O., Ohtsuka T., Nakamura M., Takenaka M. (2018). Multicenter study of early pancreatic cancer in Japan. Pancreatology.

[16] Nakahodo J., Kikuyama M., Nojiri S., Chiba K., Yoshimoto K., Kamisawa T., Horiguchi S.I., Honda G. (2020). Focal parenchymal atrophy of pancreas: An important sign of underlying high-grade pancreatic intraepithelial neoplasia without invasive carcinoma, i.e., carcinoma in situ. Pancreatology.

[17] Tanaka S., Nakao M., Ioka T., Takakura R., Takano Y., Tsukuma H., Uehara H., Suzuki R., Fukuda J. (2010). Slight dilatation of the main pancreatic duct and presence of pancreatic cysts as predictive signs of pancreatic cancer: A prospective study. Radiology.

[18] Kim S.W., Kim S.H., Lee D.H., Lee S.M., Kim Y.S., Jang J.Y., Han J.K. (2017). Isolated Main Pancreatic Duct Dilatation: CT Differentiation between Benign and Malignant Causes. AJR Am. J. Roentgenol..

[19] Basturk O., Hong S.M., Wood L.D., Adsay N.V., Albores-Saavedra J., Biankin A.V., Brosens L.A., Fukushima N., Goggins M., Hruban R.H. (2015). A Revised classification system and recommendations from the baltimore consensus meeting for neoplastic precursor lesions in the pancreas. Am. J. Surg. Pathol..

[20] Ren B., Liu X., Suriawinata A.A. (2019). Pancreatic Ductal Adenocarcinoma and Its Precursor Lesions. Am. J. Pathol..

[21] Cai J., Chen H., Lu M., Zhang Y., Lu B., You L., Zhang T., Dai M., Zhao Y. (2021). Advances in the epidemiology of pancreatic cancer: Trends, risk factors, screening, and prognosis. Cancer Lett..

[22] Wolfgang C.L., Herman J.M., Laheru D.A., Klein A.P., Erdek M.A., Fishman E.K., Hruban R.H. (2013). Recent progress in pancreatic cancer. CA Cancer J. Clin..

[23] Sagami R., Nishikiori H., Anami K., Fujiwara S., Honda K., Ikuyama S., Kitano M., Murakami K. (2018). Utility of Endoscopic Ultrasonography Screening for Small Pancreatic Cancer and Proposal for a New Scoring System for Screening. Pancreas.

[24] Matsubayashi H., Ishiwatari H., Sasaki K., Uesaka K., Ono H. (2020). Detecting Early Pancreatic Cancer: Current Problems and Future Prospects. Gut Liver.

[25] Hanada K., Okazaki A., Hirano N., Izumi Y., Teraoka Y., Ikemoto J., Kanemitsu K., Hino F., Fukuda T., Yonehara S. (2015). Diagnostic strategies for early pancreatic cancer. J. Gastroenterol..

[26] Ikemoto J., Serikawa M., Hanada K., Eguchi N., Sasaki T., Fujimoto Y., Sugiyama S., Yamaguchi A., Noma B., Kamigaki M. (2021). Clinical Analysis of Early-Stage Pancreatic Cancer and Proposal for a New Diagnostic Algorithm: A Multicenter Observational Study. Diagnostics.

[27] Zhang X.M., Mitchell D.G., Dohke M., Holland G.A., Parker L. (2002). Pancreatic cysts: Depiction on single-shot fast spin-echo MR images. Radiology.

[28] Kamata K., Kitano M., Kudo M., Sakamoto H., Kadosaka K., Miyata T., Imai H., Maekawa K., Chikugo T., Kumano M. (2014). Value of EUS in early detection of pancreatic ductal adenocarcinomas in patients with intraductal papillary mucinous neoplasms. Endoscopy.

[29] Oyama H., Tada M., Takagi K., Tateishi K., Hamada T., Nakai Y., Hakuta R., Ijichi H., Ishigaki K., Kanai S. (2020). Long-term Risk of Malignancy in Branch-Duct Intraductal Papillary Mucinous Neoplasms. Gastroenterology.

[30] Kitano M., Yoshida T., Itonaga M., Tamura T., Hatamaru K., Yamashita Y. (2019). Impact of endoscopic ultrasonography on diagnosis of pancreatic cancer. J. Gastroenterol..

[31] Moutinho-Ribeiro P., Coelho R., Giovannini M., Macedo G. (2017). Pancreatic cancer screening: Still a delusion?. Pancreatology.

[32] Sharma C., Eltawil K.M., Renfrew P.D., Walsh M.J., Molinari M. (2011). Advances in diagnosis, treatment and palliation of pancreatic carcinoma: 1990–2010. World J. Gastroenterol..

[33] Boeck S., Stieber P., Holdenrieder S., Wilkowski R., Heinemann V. (2006). Prognostic and therapeutic significance of carbohydrate antigen 19-9 as tumor marker in patients with pancreatic cancer. Oncology.

[34] Fahrmann J.F., Schmidt C.M., Mao X., Irajizad E., Loftus M., Zhang J., Patel N., Vykoukal J., Dennison J.B., Long J.P. (2021). Lead-Time Trajectory of CA19-9 as an Anchor Marker for Pancreatic Cancer Early Detection. Gastroenterology.

[35] Van Manen L., Groen J.V., Putter H., Vahrmeijer A.L., Swijnenburg R.J., Bonsing B.A., Mieog J.S.D. (2020). Elevated CEA and CA19-9 serum levels independently predict advanced pancreatic cancer at diagnosis. Biomarkers.

[36] Liu J., Gao J., Du Y., Li Z., Ren Y., Gu J., Wang X., Gong Y., Wang W., Kong X. (2012). Combination of plasma microRNAs with serum CA19-9 for early detection of pancreatic cancer. Int. J. Cancer.

[37] Kirkegård J., Cronin-Fenton D., Heide-Jørgensen U., Mortensen F.V. (2018). Acute Pancreatitis and Pancreatic Cancer Risk: A Nationwide Matched-Cohort Study in Denmark. Gastroenterology.

[38] Munigala S., Kanwal F., Xian H., Scherrer J.F., Agarwal B. (2014). Increased risk of pancreatic adenocarcinoma after acute pancreatitis. Clin. Gastroenterol. Hepatol..

[39] Kirkegård J., Mortensen F.V., Cronin-Fenton D. (2017). Chronic Pancreatitis and Pancreatic Cancer Risk: A Systematic Review and Meta-analysis. Am. J. Gastroenterol..

[40] Permuth-Wey J., Egan K.M. (2009). Family history is a significant risk factor for pancreatic cancer: Results from a systematic review and meta-analysis. Fam. Cancer.

[41] Klein A.P., Brune K.A., Petersen G.M., Goggins M., Tersmette A.C., Offerhaus G.J., Griffin C., Cameron J.L., Yeo C.J., Kern S. (2004). Prospective risk of pancreatic cancer in familial pancreatic cancer kindreds. Cancer Res..

[42] Silverman D.T., Schiffman M., Everhart J., Goldstein A., Lillemoe K.D., Swanson G.M., Schwartz A.G., Brown L.M., Greenberg R.S., Schoenberg J.B. (1999). Diabetes mellitus, other medical conditions and familial history of cancer as risk factors for pancreatic cancer. Br. J. Cancer.

[43] Howes N., Lerch M.M., Greenhalf W., Stocken D.D., Ellis I., Simon P., Truninger K., Ammann R., Cavallini G., Charnley R.M. (2004). Clinical and genetic characteristics of hereditary pancreatitis in Europe. Clin. Gastroenterol. Hepatol..

[44] Ben Q., Xu M., Ning X., Liu J., Hong S., Huang W., Zhang H., Li Z. (2011). Diabetes mellitus and risk of pancreatic cancer: A meta-analysis of cohort studies. Eur. J. Cancer.

[45] Yuan C., Babic A., Khalaf N., Nowak J.A., Brais L.K., Rubinson D.A., Ng K., Aguirre A.J., Pandharipande P.V., Fuchs C.S. (2020). Diabetes, Weight Change, and Pancreatic Cancer Risk. JAMA Oncol..

[46] Sadr-Azodi O., Gudbjörnsdottir S., Ljung R. (2015). Pattern of increasing HbA1c levels in patients with diabetes mellitus before clinical detection of pancreatic cancer—A population-based nationwide case-control study. Acta Oncol..

[47] Sah R.P., Nagpal S.J., Mukhopadhyay D., Chari S.T. (2013). New insights into pancreatic cancer-induced paraneoplastic diabetes. Nat. Rev. Gastroenterol. Hepatol..

[48] Lugo A., Peveri G., Bosetti C., Bagnardi V., Crippa A., Orsini N., Rota M., Gallus S. (2018). Strong excess risk of pancreatic cancer for low frequency and duration of cigarette smoking: A comprehensive review and meta-analysis. Eur. J. Cancer.

[49] Izumi Y., Hanada K., Okazaki A., Minami T., Hirano N., Ikemoto J., Kanemitsu K., Nakadoi K., Shishido T., Katamura Y. (2019). Endoscopic ultrasound findings and pathological features of pancreatic carcinoma in situ. Endosc. Int. Open.

[50] Recavarren C., Labow D.M., Liang J., Zhang L., Wong M., Zhu H., Wang J., Francis F., Xu R. (2011). Histologic characteristics of pancreatic intraepithelial neoplasia associated with different pancreatic lesions. Hum. Pathol..

[51] Maire F., Couvelard A., Palazzo L., Aubert A., Vullierme M.P., Rebours V., Hammel P., Sauvanet A., Levy P., Ruszniewski P. (2013). Pancreatic intraepithelial neoplasia in patients with intraductal papillary mucinous neoplasms: The interest of endoscopic ultrasonography. Pancreas.

[52] Nehra D., Oyarvide V.M., Mino-Kenudson M., Thayer S.P., Ferrone C.R., Wargo J.A., Muzikansky A., Finkelstein D., Warshaw A.L., Castillo C.F. (2012). Intraductal papillary mucinous neoplasms: Does a family history of pancreatic cancer matter?. Pancreatology.

[53] Sharma A., Kandlakunta H., Nagpal S.J.S., Feng Z., Hoos W., Petersen G.M., Chari S.T. (2018). Model to Determine Risk of Pancreatic Cancer in Patients with New-Onset Diabetes. Gastroenterology.

[54] Klein A.P., Lindström S., Mendelsohn J.B., Steplowski E., Arslan A.A., Bueno-de-Mesquita H.B., Fuchs C.S., Gallinger S., Gross M., Helzlsouer K. (2013). An absolute risk model to identify individuals at elevated risk for pancreatic cancer in the general population. PLoS ONE.

[55] Dong X., Lou Y.B., Mu Y.C., Kang M.X., Wu Y.L. (2018). Predictive Factors for Differentiating Pancreatic Cancer-Associated Diabetes Mellitus from Common Type 2 Diabetes Mellitus for the Early Detection of Pancreatic Cancer. Digestion.

[56] Boursi B., Finkelman B., Giantonio B.J., Haynes K., Rustgi A.K., Rhim A.D., Mamtani R., Yang Y.X. (2017). A Clinical Prediction Model to Assess Risk for Pancreatic Cancer Among Patients with New-Onset Diabetes. Gastroenterology.

[57] Sakamoto H., Harada S., Nishioka N., Maeda K., Kurihara T., Sakamoto T., Higuchi K., Kitano M., Takeyama Y., Kogire M. (2017). A Social Program for the Early Detection of Pancreatic Cancer: The Kishiwada Katsuragi Project. Oncology.

